# An anti-PF4 antibody-related disorder with cerebral venous sinus thrombosis and thrombocytopenia initially presenting as intracranial hemorrhage

**DOI:** 10.1007/s00415-024-12373-6

**Published:** 2024-04-10

**Authors:** Matthias Wittstock, Daniel Cantré, Sae-Yeon Won, Alexandra V. Jürs, Jan Wesche, Nico Greger, Andreas Greinacher, Thomas Thiele

**Affiliations:** 1 Department of Neurology, University Medical Center Rostock, Schillingallee 36, 18057 Rostock, Germany; 2 Institute of Diagnostic and Interventional Radiology, Pediatric Radiology and Neuroradiology, University Medical Center Rostock, Rostock, Germany; 3 Department of Neurosurgery, University Medical Center Rostock, Rostock, Germany; 4 Translational Neurodegeneration Section “Albrecht Kossel”, Department of Neurology, University Medical Center Rostock, Rostock, Germany; 5 Institute of Transfusion Medicine, University Medical Center Greifswald, Greifswald, Germany; 6 Institute of Transfusion Medicine, University Medical Center Rostock, Rostock, Germany

Dear Sirs,

Anti-platelet factor (PF4) disorders gained major attention worldwide, when anti-PF4 IgG antibodies were identified as cause of vaccine-induced immune thrombotic thrombocytopenia (VITT) after adenoviral vector-based vaccines [1]. VITT occurs 5–20 days after vaccination and manifests with venous and arterial thrombosis at unusual locations such as splanchnic or most often as cerebral venous sinus thrombosis (CVST) [2]. About 8% of the patients with CSVT have thrombocytopenia, and VITT-like anti-PF4 antibodies occur even without prior vaccination [2–4]. Especially adenovirus infections can trigger these antibodies. These observations suggest that anti-PF4-related “VITT-like” disorders may be more common than previously anticipated. Herein, we present such a patient highlighting the importance of early clinical recognition of anti-PF4 VITT-like disorders.

A 57-year-old female patient was transferred to our department with a 1-week-history of fluctuating headache und a 3-day-lasting rectal bleeding. She had a history of sigma diverticulosis, IgA deficiency syndrome and nicotine abuse. No neoplasms, infectious diseases or head trauma were reported. She was vaccinated against Covid-19 with mRNA-based vaccines 24 month ago. Cranial computed tomography (CT) from the external hospital displayed a small, atypical left-sided intracerebral hemorrhage (ICH). Moreover, a “hyperdense triangle sign” of the left transverse and sigmoid sinus suggestive for CSVT (Fig. 1A) was present, but had been overlooked initially.

**Fig. 1:**
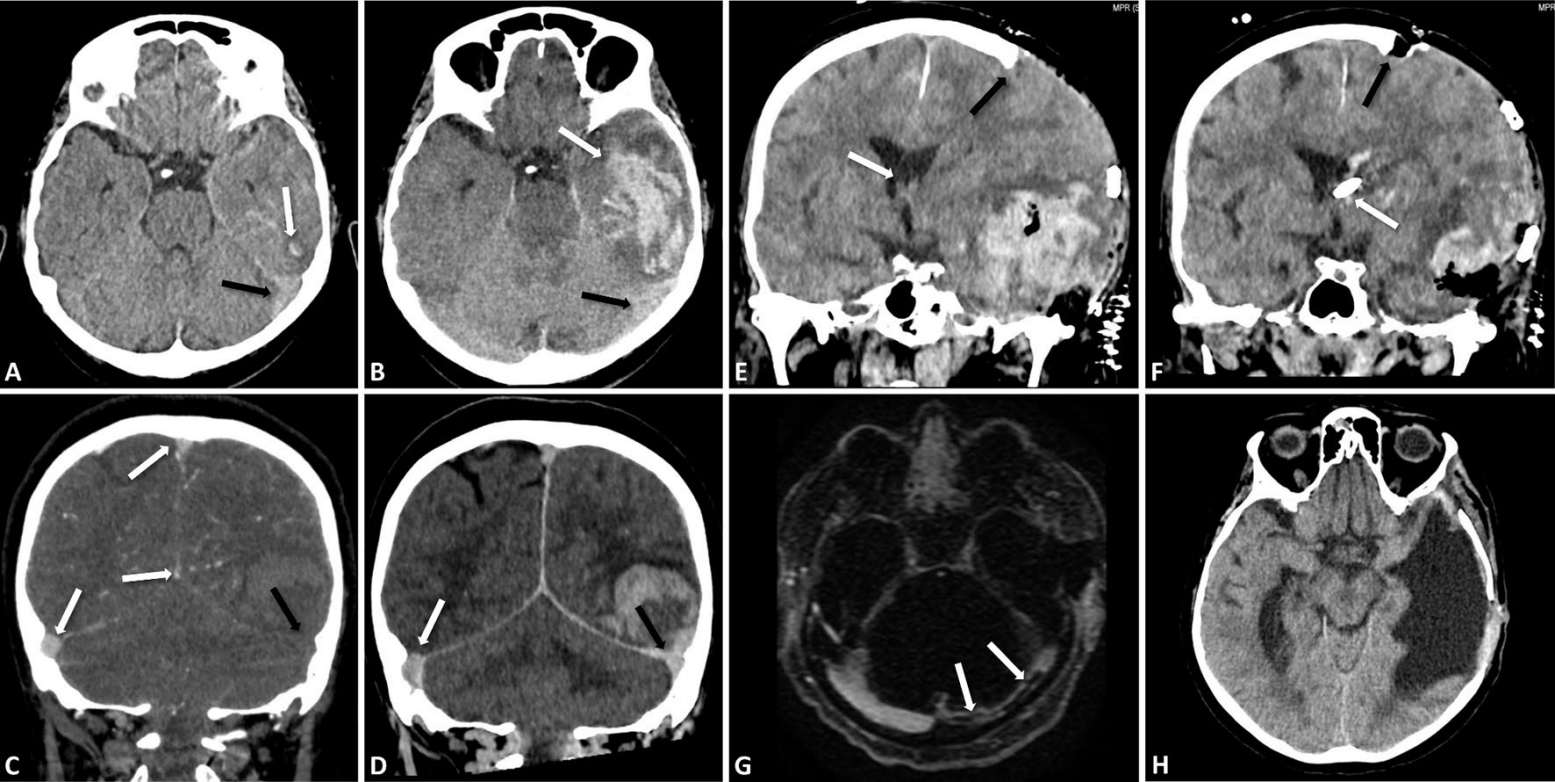
Representative cranial CT (**A–F**, **H**) and MRI (**G**) images throughout the therapeutic course. **A** Axial slice of the initial cranial CT on the day of admission, with small intracerebral hemorrhage (ICH, white arrow) and mild adjacent subarachnoid hemorrhage (SAH) in the left temporal lobe, as well as discrete “hyperdense triangle sign” of the left transverse sinus (black arrow), suggestive for sinus thrombosis. **B** Corresponding axial slice of follow-up CT 3 h later with severe progression of left temporal ICH (white arrow) with mass effect, and SAH, and persistent discrete hyperdensity of the left transverse sinus (black arrow), as well as discrete adjacent subdural hemorrhage (SDH). **C** Coronal slice of the CT-angiography in an arterial phase, acquired immediately after **B** no signs of aneurysm, arteriovenous malformation or dural arteriovenous fistula. Superior and inferior sagittal sinus, as well as right transverse sinus are already contrasted (white arrows), whereas contrast is missing in the left transverse sinus (black arrow) in the arterial phase, again, suspicious for left-sided sinus thrombosis. In the post-venous phase 30 min later, **D** there is symmetric contrast enhancement of the somewhat hypoplastic and non-dilated left transverse and sigmoid sinus (black arrow) when compared to the right side (white arrow), ruling against acute occlusive sinus thrombosis. Left-sided cortical veins were difficult to assess because of mass effect, SAH and SDH, but seemed mainly contrasted. **E** After acute decompressive craniectomy and hematoma evacuation on the day of admission, coronal reformation of the postoperative CT shows recurrent mass effect of relapsing left temporal ICH and occlusive hydrocephalus with relevant midline shift to the right (white arrow) and compression of the cortex at the edge of the craniectomy (black arrow), indicating insufficient decompression. Corresponding coronal slice **F** of CT after operative revision on the following day with extended craniectomy (black arrow), repeat hematoma evacuation and implantation of an external ventricular drain in the frontal horn of the left lateral ventricle (white arrow), shows sufficient decompression. An axial reconstruction of a dynamic contrast-enhanced MRI angiography in the venous phase **G** on day 8 shows non-occlusive thrombosis of the hypoplastic left transverse sinus (white arrows), reaching from the torcula to the jugular foramen. Axial slice of the final CT **H** after bone flap reinsertion 4 months later with large porencephalic defect of the left temporal lobe after complete resorption of the hemorrhage, and no further complications

After admission, she showed clinically deterioration of consciousness, severe aphasia, and a mild left-sided brachiocephalic hemiparesis. Laboratory testing revealed a low platelet count (47 × 10^9^/l), a hemoglobin level of 12.1 g/dl, and normal hematocrit. Routine coagulation parameters were normal, but d-dimers were highly elevated with 35 mg/l FEU [< 0.5 mg/l FEU]. Infectious disease testing including SARS-CoV-2 was negative.

Immediate cranial CT follow-up showed severe progression of the left-sided ICH accompanied by subarachnoidal (SAH) and subdural hemorrhages on the same side (Fig. 1B). Further, CT-angiography (CTA) recorded only an arterial contrast-enhanced phase, in which intracranial arterial aneurysm, arteriovenous malformation and dural arteriovenous fistula were ruled out. Venous sinuses of the midline and right side were contrasted unlike the left transverse sinus and sigmoid sinuses which were not contrasted (Fig. 1C). However, the next cranial CT 30 min after contrast medium injection, the left transverse and sigmoid sinuses as well as the large cortical veins showed normal contrast enhancement in the post-venous phase when compared to the right hemisphere (Fig. 1D). At that time, CVST could not be ruled out because reduced venous return from the drainage area (reduced venous return from the hemorrhagic tissue that is no longer perfused plus space-occupying effect) might be an alternative explanation for the altered venous drainage pattern.

Progressive SAH and intraventricular hemorrhage worsened by severe left hemispheral perihematomal edema prompted left-sided decompression hemicraniectomy (Fig. 1E). A revision was necessary with an enlargement of the hemicraniectomy, hematoma evacuation and insertion of an external ventricular drainage due to cerebrospinal fluid congestion and further bleeding progression (Fig. 1F). Packed red cells and platelet concentrates were administered intraoperatively and during the course of intensive care treatment.

On day 3, platelet counts (15 × 10^9^/l) and hemoglobin levels (6.6 g/dl) further declined. While gastroscopy and colonoscopy did not show any bleeding, thoracic CT scan revealed bilateral peripheral and central pulmonary embolism on day 4 instead (not shown). Concomitant thrombosis and persisting thrombocytopenia while on thromboprophylaxis with low molecular weight heparin (LMWH) prompted testing for heparin-induced thrombocytopenia (HIT) and other potential causes of prothrombotic thrombocytopenia syndromes (fibrinogen 4.9 g/l, d-dimer 33 mg/l FEU; ADAMTS 13-activity 52.0%; ADAMTS 13 antigen 0.42 IU/ml; antiphospholipid antibodies negative). The PF4/heparin ELISA was strongly positive, and the negative result of the functional heparin-induced platelet activation (HIPA) assay made HIT very unlikely. However, the functional assay for VITT-like antibodies, in the presence of PF4 (PIPA), was strongly positive, typical for an anti-PF4-IgG-related thrombosis and thrombocytopenia [5]. In addition, we confirmed IgG antibodies recognizing PF4 only but not anti-PF4/heparin antibodies by an experimental assay [4].

Anticoagulation was instantly switched from LMWH to therapeutic dose argatroban. Thereafter, platelet counts started to improve at day 9 and reached normal levels at day 11 (Fig. 2). Due to the history of IgA deficiency, no intravenous immunoglobulins (IVIG) were administered, as suggested by Lindhoff-Last et al. [5]. IVIG treatment can induce severe anaphylaxis in patients with IgA deficiency [6]. Furthermore, plasmapheresis and immunosuppressive treatment were omitted because the prothrombotic coagulopathy could be controlled by non-heparin anticoagulation alone after cranial bleeding and craniectomy. The further clinical course was complicated by intracranial vasospasm, nosocomial pneumonia, prolonged weaning and delayed awakening.

**Fig. 2:**
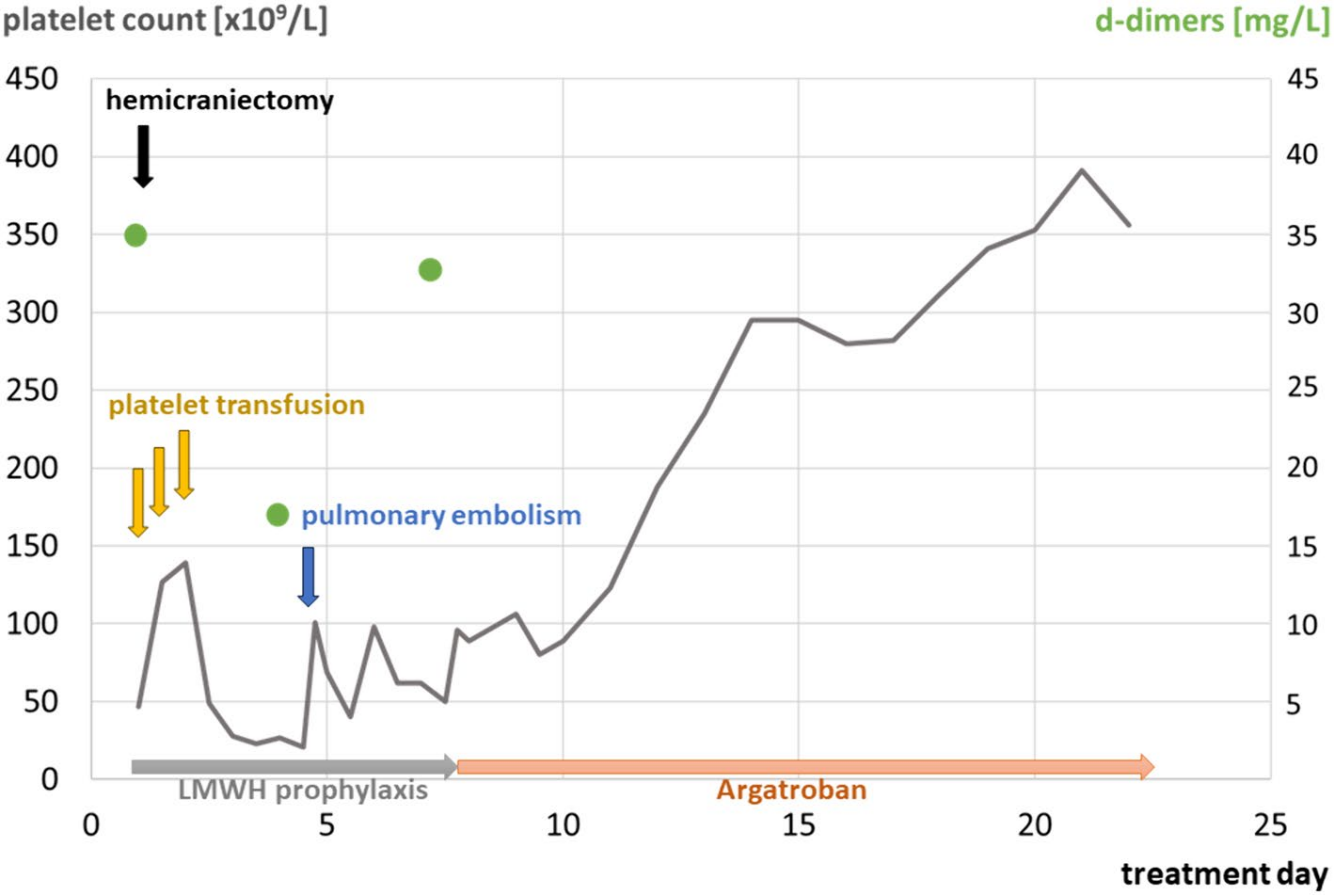
Clinical course and evolution of platelet counts during the first hospital stay. Day of admission (day 1). Initial platelet counts were low, but d-dimers were highly elevated. Platelet concentrates were transfused to facilitate hemicraniectomy, but platelet counts decreased rapidly thereafter. On day 4, pulmonary embolism occurred prompting differential diagnosis of thrombosis and thrombocytopenia. When anticoagulation was switched from LMWH to argatroban, low platelet counts steadily resolved

Contrast-enhanced cranial magnetic resonance imaging (MRI) on day 8 confirmed the non-occlusive thrombi in the hypoplastic transverse and sigmoid sinus on the left side, starting at the torcula (Fig. 1G). The last cranial CT scan in our center at day 20 demonstrated the hematoma resorption in a timely manner. Anticoagulation was switched from argatroban to direct oral anticoagulation with apixaban 2 × 2.5 mg (reduced dose to avoid recurrent hemorrhage).

After rehabilitation, the patient was admitted again at day 100 for planned bone flap reinsertion (Fig. 1H). Clinically, the patient improved showing mild motor aphasia and only slight hemiparesis. Four months later, platelet counts were normal and anti-PF4-antibodies no longer detectable.

We report here a VITT-like anti-PF4 disorder in a patient with ICH and unexplained pulmonary embolism and thrombocytopenia.

In case of an atypical localization of the hematoma, CVST needs to be considered. The situation becomes challenging when platelet counts are low at the same time. While low platelet counts alone can increase the risk of bleeding, platelet counts in this case were initially too high to explain ICH and unlikely the cause of bleeding. Furthermore, d-dimers were highly elevated which indicates an underlying coagulopathy. However, persisting thrombocytopenia (Fig. 2) and concomitant administration of LMWH raised initially the suspicion of HIT. However, extended laboratory testing excluded HIT but unraveled an anti-PF4 antibody-related disorder. As previously reported, the anti-PF4 antibodies are known to cause severe prothrombotic disease in conjunction with thrombocytopenia [5]. Interestingly, we found anti-PF4 antibodies to be only transiently present, as it is typical also for HIT and VITT [7, 8].

CVST is a challenging diagnosis, particularly based on a blanco CT affecting 10–20% of patients with later proven CVST [9]. CT misdiagnosis is more likely if sinuses are suppressed by hemorrhage edema or by congestion due to reduced return of blood from the drainage area (reduced venous return from the hemorrhagic tissue that is no longer perfused plus space-occupying effect). Radiological findings were immediately reassessed in light of the positive anti-PF4 IgG tests and a follow-up cranial MRI was performed, which finally established the diagnosis of CVST as the most likely cause for secondary ICH. This is a typical finding also in anti-PF4 antibody-mediated VITT and VITT-like disorders [5, 10].

PF4 antibody-related disorders are severe and their diagnosis may be challenging. ICH could be the first presentation but usually develops secondary to CVST. When patients with unclear thrombosis, thrombocytopenia and highly elevated d-dimers present with an ICH, immediate radiological workup should be arranged to establish or rule out an underlying CVST. Laboratory testing for anti-PF4 antibodies is strongly recommended even if no heparin or vector-based vaccination has been given prior to symptom onset. For anti-PF4 antibody testing, a microtiter plate assay must be used. Rapid tests do not recognize anti-PF4 antibodies but only anti-PF4/heparin antibodies. The functional PIPA test can finally secure the diagnosis. When anti-PF4 antibodies are detected, alternative non-heparin anticoagulation should be immediately started possibly in combination with intravenous immunoglobulins to treat anti-PF4-related disorder and to reduce the risk of additional thromboses [11].

## Data Availability

Deidentified patient data will be shared upon reasonable requests.
